# The role of carcinoembryonic antigen as an assessment tool for predicting disease severity among patients with colorectal cancer in resource-poor setting of Kwazulu-Natal, South Africa

**DOI:** 10.11604/pamj.2021.39.185.20520

**Published:** 2021-07-08

**Authors:** Yugan Dylan Naicker, Zaheer Moolla, Thandinkosi Madiba

**Affiliations:** 1 Department of Surgery, School of Clinical Medicine, College of Health Sciences, University of KwaZulu-Natal, Durban, South Africa

**Keywords:** Colorectal cancer, carcinoembryonic antigen, sub-Saharan Africa

## Abstract

**Introduction:**

the most reliable screening tool for colorectal cancer, colonoscopy, is not readily accessible in resource-deprived settings of KwaZulu-Natal. The aim of this study was to determine whether serum carcinoembryonic antigen (CEA) levels in patients symptomatic for lower gastrointestinal (GI) pathology correlates with the histological presence and severity of primary colorectal cancer in a large referral centre. Perhaps CEA may have a larger role as a marker for colorectal cancer (CRC) development in these resource deprived communities.

**Methods:**

this study was a retrospective analysis of prospectively collected clinical data of 380 pretreatment patients with colorectal cancer attending a tertiary referral centre in KwaZulu-Natal. Data were analyzed using descriptive statistics and findings were compared with those from the existing literature.

**Results:**

the mean CEA level of the study population was 170.0 ± 623.3 μg/l. The number of participants with a CEA level <5 μg/l was 151 (39.74%) whilst the majority 229 (60.26%) had a CEA level ≥ 5 μg/l. There was no significant correlation between CEA levels and gender (p=0.8) or age (p=0.6). CEA levels were highest in the black African race group. Pairwise comparison demonstrated a statistically significant difference between the black and Indian population groups (p=0.02). The current study demonstrates an upregulation of CEA as the stage of CRC progresses (p<0.0001).

**Conclusion:**

there was no significant difference in CEA levels across age and gender. A positive correlation was noted between CEA level and stage of CRC. Carcinoembryonic antigen levels were highest in the black race group. Low sensitivity of CEA as a screening test for CRC was confirmed.

## Introduction

Colorectal cancer (CRC) is the third most common malignancy in the world [1]. The global incidence of CRC is projected to rise up to 60% by 2030 [2,3]. More than one million new cases are diagnosed per annum and 530,000 deaths are reported per year. Colorectal cancer evolves across four distinct carcinogenic conduits: the chromosomal instability pathway [4], the microsatellite instability pathway [4], the cytosine-phosphate-guanine (CpG) methylator pathway-1 [5] and pathway-2 [6]. Importantly the slow growth of CRC warrants early screening to reduce both the incidence of and mortality from the disease. The aim of screening should be to detect CRC at a relatively early stage (stage 1 and 2). However, currently the most reliable diagnostic tool, namely colonoscopy is not easily accessible in resource-deprived settings.

Carcinoembryonic antigen (CEA) is one of the most widely used tumour markers worldwide. It was first described in 1965 by Gold and Freedman [7,8]. Carcinoembryonic antigen is a glycoprotein with a molecular weight of 200,000 [9]. It was initially identified and immunolocalized on both fetal colon and colon adenocarcinoma [10]. Carcinoembryonic antigen has been found to be overexpressed in a wide variety of epithelial malignancies [11]. The literature attributes the elevated CEA levels in CRC to tumour vascularity, necrosis, mitotic activity and differentiation [11]. It is thus widely used clinically as both a blood and tissue tumour marker of epithelial malignancy, especially for tumours of the colon and rectum. Carcinoembryonic antigen is also present in normal tissue at levels of ≤3 ng/ml, which is 60-fold lower concentration than that seen in malignant tissue [12].

Several studies have implicated high preoperative concentrations of CEA with adverse outcome in patients with Duke´s B colorectal cancer [13]. More recently, Su *et al*. [14] demonstrated an overall sensitivity of CEA for the detection of CRC at 37.0%; however, they also found levels to be directly related to stage namely 21.4%, 38.9% and 41.7% for stages I-III, respectively. These are unacceptably low predictive values, hence the need to rely on colonoscopy as a gold standard. Data on the role of CEA as a prognostic marker for CRC in sub-Saharan Africa are limited [15].

KwaZulu-Natal is a province of South Africa, with an estimated population of 12 million. Despite this large population, only 8 medical facilities offer colonoscopy to the public health care sector, with resultant huge delays in acquiring screening or diagnostic colonoscopies in this resource-deprived setting. Currently, a reliable tumour marker for CRC is unavailable.

In light of the fact that CRC is the fourth most common cancer in South Africa (SA) [16], the aim of this study was to determine whether serum CEA levels in patients symptomatic for lower GI pathology correlate with the histological presence and severity of primary colorectal cancer in a large referral centre within KwaZulu-Natal. Perhaps CEA may have a larger role as a risk assessment tool for the development of CRC in these resource-deprived communities. However thus far, the value of CEA in screening, diagnosis and prognosis in KwaZulu-Natal remains to be accurately defined.

## Methods

**Study setting:** this study was carried out in the Colorectal Clinic at Inkosi Albert Luthuli Central Hospital, the tertiary referral hospital in Durban, South Africa.

**Study population:** the study population (n=380) consisted of patients diagnosed with colorectal cancer who were extracted from the on-going colorectal cancer database which is archived in the Gastrointestinal Cancer Research Centre of the University of KwaZulu-Natal. Patients who were tested for baseline CEA form the basis of this analysis. They included Indian, black African, white and coloured patients, as described by the South African government. Carcinoembryonic antigen levels were compared across various age groups and both genders with colorectal cancer. International Statistical Classification of Diseases and Related Health Problems (10^th^ revision) (ICD-10) diagnosis codes were used to identify colorectal cancer.

**Study design:** this was a retrospective analysis of prospectively collected clinical data of patients with known baseline CEA levels. Serum was collected from patients with confirmed colorectal cancer but who have not yet had resectional surgery. The period of study was 2007 to 2016. The serum was immediately analysed for CEA levels using the enzyme-linked immunosorbent assay (method). A serum CEA >5 μg/l was considered elevated. Colonoscopy was performed by gastroenterologists using an Olympus Medical Systems, Tokyo, Japan colonoscope. All participants received four litres of polyethylene glycol solution for bowel preparation. Biopsies were obtained and evaluated by state pathologists via histopathological examination. Tumor, nodes and metastase (TNM) and International Union Against Cancer (UICC) staging are used in the colorectal cancer database. For the purpose of this paper UICC staging was used. The inclusion criteria for the study comprised patients with histologically confirmed colorectal cancer and staging. Demographics, site of primary tumour, presence of metastatic disease and CEA level prior to chemotherapy, radiotherapy and/or definitive surgery were collated into a datasheet for statistical analysis.

**Ethical considerations:** this retrospective clinical study received institutional ethics approval (BE016/17). Additionally, approval was obtained from the National Department of Health and the Hospital manager.

**Statistical analysis:** nonparametric data are represented as median and interquartile range (IQR). GraphPad Prism 5.00 for Windows (GraphPad Software, San Diego, California, USA) was used for data analysis. The Fisher´s Exact/Chi-Square test was used for analysis. To determine statistical significance across all study groups a Kruskal-Wallis (Dunn´s multiple comparison) or a Mann-Whitney U test was carried out. Spearman coefficients were used to correlate CEA levels with the patient demographic as well as the stage of CRC. A p value of <0.05 was considered as statistically significant.

## Results

There was a total of 380 patients with colorectal cancer including 46 (12.11%) younger presenters (age <40 years) and 334 (87.89%) older presenters (age <40 years). The study population consisted of 380 patients with a histologically confirmed colorectal cancer. Two hundred and twelve (55.8%) participants were males and 168 (44.2%) were female (male: female ratio = 1: 0.79). The mean age of the study population was 57.7 ± 13.6 years.

Carcinoembryonic antigen levels were stratified into <5 μg/l and ≥5 μg/l and is outlined in Table 1. The mean CEA level of the study population was 170.0 ± 623.3 μg/l. The number of participants with a CEA level <5 μg/l was 151 (39.74%) whilst 229 (60.26%) had a CEA level ≥5 μg/l.

**Table 1:** carcinoembryonic antigen level μg/l based on race, age and gender

	Sample size (n)	CEA level*	p value
**Race**			
Indian	181	155.8 ± 612.9	p=0.08 #
Black African	131	232.0 ± 742.9	
Coloured	12	98.98 ± 349.7	
White	56	39.66 ± 104.8	
**Age**			
<40 Years	46	266.7 ± 736.3	p=0.60
>40 Years	334	157.5 ± 607.5	
**Gender**			
Female	168	219.2 ± 779.0	p=0.80
Male	212	131.1 ± 462.7	

Legend:* CEA level in mean + standard deviation; # pairwise comparisons of each race groups vs another one, as well as black African vs all others (Mann-Whitney Test): between all 4 races; p=0.08, black African vs Indian p=0.04; black African vs coloured p=0.06; black African vs white p=0.13; black African vs all others p=0.02; Indian vs coloured p=0.23; Indian vs white p=0.93

Carcinoembryonic antigen levels were evaluated across the study population and there was no significant difference between a CEA level ≥5 μg/l and gender (Kruskal-Wallis H=272.8; p=0.8 (Table 1)). Carcinoembryonic antigen levels were higher in females (219.2 ± 77.9 μg/l) compared to males (131.1± 462.7 μg/l). However, this did not reach statistical significance.

Carcinoembryonic antigen levels were highest in black African patients (232.0 ± 742.9 μg/l) compared to other population groups (Table 1). The difference across the population groups did not reach statistical significance (p=0.08). However, pairwise comparison demonstrated a statistically significant difference between the black African and Indian population groups (p=0.04). The difference in the CEA levels between the black African group on the one hand and the other population groups combined on the other, was also statistically significant (p=0.02).

**Figure 1:**
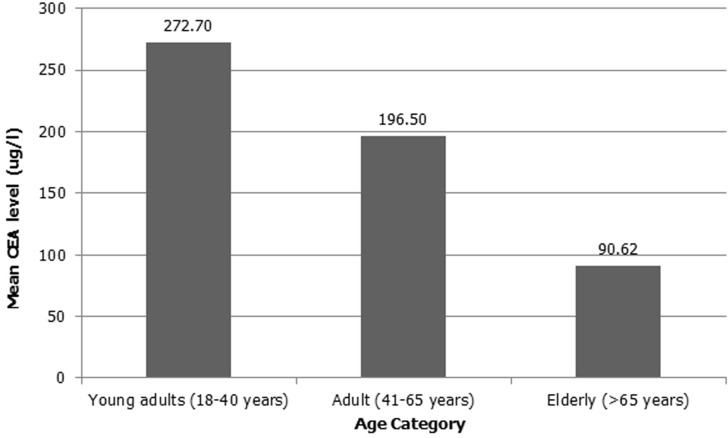
correlation between the level of CEA (μg/l) and age (r^2^=0.008; p=0.008), mean CEA levels declined as age increased

**Figure 2:**
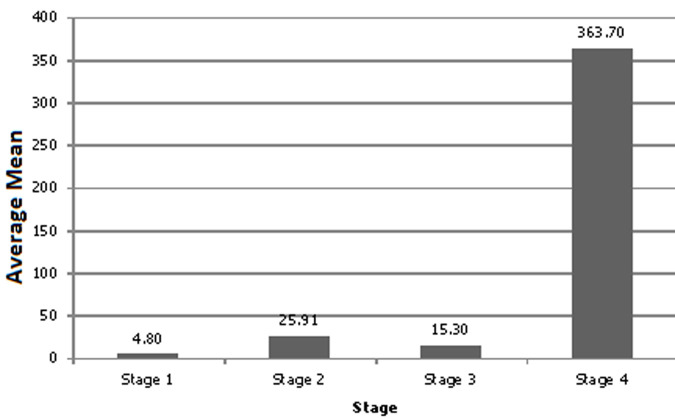
correlation between the level of CEA (μg/l) and the stage of CRC (r^2^=0.054; p<0.0001), an elevated CEA level is associated with a higher stage of CRC

As shown in Figure 1, mean CEA levels declined with increasing age (p=0.008). There was no significant difference in CEA levels between young presenters (age <40 years) and older presenters (age >40 years) (p=0.87). This is well demonstrated in Table 2. Additionally, as the severity of CRC increased, CEA levels increased significantly across all age groups (Figure 2). Table 3 shows the staging in study patients. As shown in Figure 2, an elevated CEA level is associated with a higher stage of CRC (r^2^=0.054; p<0.0001). The odds ratio of having a CEA level ≥5 μg in stage 4 CRC was 11.28 (CI= 4.51 - 28.18; p<0.0001).

**Table 2:** contingency of CEA level (μg/l) in gender, age and race

Gender										
CEA Level (μg/l)	Male (n=212)				Female (168)					Fisher's exact test
	N		%		N			%		p = 0.75
<5	86		40.56		65			38.69		
>5	126		59.43		103			61.31		
**Age**										
**CEA Level (μg/l)**	**< 40 years (n=46)**				**> 40 years (n=334)**					**Fisher's exact test**
	**N**		**%**		**N**			**%**		**p = 0.87**
<5	18		39.13		135			40.42		
>5	28		60.87		199			59.58		
**Race**										
**CEA Level (μg/l)**	**Indian (n=181)**		**Black African (n=131)**		**White (n=56)**			**Coloured (n=12)**		**Chi square test**
	**N**	**%**	**N**	**%**	**n**	**%**		**N**	**%**	**X2=2.90,3 p=0.40**
<5	75	41.44	45	34.35	25	44.64		6	50	
>5	106	58.57	86	65.65	31	55.36		6	50	
										

**Table 3:** staging in 380 patients with colorectal cancer and known preoperative CEA levels

Stage	N	%
Stage I	24	9
Stage II	59	15.5
Stage III	58	15.3
Stage IV	143	37.6
Not staged	86	22.6

## Discussion

The current study demonstrates an upregulation of CEA as the stage of CRC progresses. Whereas age is an established risk factor for the development of CRC [17], we report in this study that there was no significant difference between the CEA levels and age. This is supported by a large Korean study, which showed serum CEA to be a vital risk factor for the development of advanced colorectal neoplasms in both young (<50 years) and old adults (≥50 years) [17].

Internationally the incidence of CRC is reportedly higher in men than in women and strongly increases with age [18]. In South Africa, the cumulative lifetime risk of developing CRC amongst all race groups is reported at 1.24 for males and 0.74 for females [16]. This series demonstrated no significant difference in the CEA levels between males and female genders.

The current study reports a significant positive correlation between baseline CEA levels and the stage of CRC. The clinical implication of this finding is that CEA levels may be used as a prognostic marker for stage IV CRC although it does not appear to be a good marker for stage I, II and III disease. However, the clinical use of CEA as a prognostic marker for stage IV CRC is limited as management at this stage of disease would largely be palliative. These findings are corroborated by the findings of Kim *et al*. which demonstrated that elevated CEA levels correlated well with advanced stages of CRC and a poorer clinical outcomes [17].

Zhang *et al*. have previously postulated that the combined detection of serum CEA and CA 19-9 could play a complementary role in the diagnosis of CRC and could significantly improve the sensitivity for the diagnosis of CRC [19]. CA 19-9 might be a tumour biomarker in addition to CEA for CRC [19]. Despite this proposal, we demonstrated in this series that only 60.26% of patients with confirmed CRC had an elevated CEA level. Other authors have also shown a very low sensitivity of this test and concluded that there is no role for CEA assessment as a screening tool for CRC [20]. We concede that CEA has no diagnostic role due to its low sensitivity and specificity to CRC. Therefore, in light of the poor access and long delays associated with colonoscopy in the public health care sector of South Africa [21], it is crucial that one is able to better evaluate patients with suspected CRC via non-invasive techniques such as the stool guaiac occult blood test.

Limitations of the present study include a lack of quantitative stratification of groups into smoking and non-smoking sub-groups bearing in mind that CEA concentrations are affected by a variety of factors including smoking and gender [22]. Also, comparison of the white or coloured race groups was not statistically significant due to the small sample sizes of these groups respectively. Furthermore, a potential bias of the sampling technique is that this study only included patients who had a pre-operative level of CEA. Also, patients are often referred to the colorectal unit after surgical resection with pre-cluded their inclusion in this study.

## Conclusion

This study reports no significant difference in CEA levels across age or gender. The CEA levels were found to be highest in the black African race group and the present series confirms a low sensitivity of CEA as a diagnostic test for CRC. Finally, this study does identify that CEA levels are higher with increased stage of CRC. The clinical implication of this finding is that CEA levels may be a reasonably good marker for stage IV CRC although it has failed to demonstrate reliability as a risk assessment tool for stage I, II and III disease. Having said this, we concede that the clinical use of CEA as a marker even for stage IV CRC remains limited as management of this stage of disease would largely be palliative. We have therefore not shown the benefit of CEA levels as a risk assessment tool to be used to fast-track symptomatic patients for colonoscopy. For now it is worth exploring the possibility of fast-tracking patients with high CEA to radiological investigations and if extensive metastatic disease is found, then a possibility of colorectal cancer can be entertained and thus the need for urgent diagnostic colonoscopy avoided. We further recommend that less invasive approaches be promoted in resource-limited settings including the use of guaiac faecal occult blood test (gFOBT). Special attention to improve colorectal cancer screening in African countries are necessary to improve survival rates. Finally, the role of hereditary colon cancer in young black Africans and its impact on survival remains largely unexplored.

**Funding:** College of Health Sciences, University of KwaZulu-Natal provided funding for this project.

### What is known about this topic

- Carcinoembryonic antigen has a low sensitivity as a diagnostic test for CRC;
- Access to diagnostic colonoscopy for CRC is limted in the public health care sector of South Africa.

### What this study adds

- Carcinoembryonic antigen levels were found to be highest in the black African race group;
- Carcinoembryonic antigen levels may be a reasonably good marker for stage IV CRC;
- There is no benefit of using CEA levels as a tool to fast-track symptomatic patients for colonoscopy.
